# Stabilization of a Membrane-Associated Amyloid-β Oligomer for Its Validation in Alzheimer's Disease

**DOI:** 10.3389/fmolb.2018.00038

**Published:** 2018-04-19

**Authors:** Montserrat Serra-Batiste, James Tolchard, Fabrice Giusti, Manuela Zoonens, Natàlia Carulla

**Affiliations:** ^1^Institute for Research in Biomedicine (IRB Barcelona), The Barcelona Institute Science and Technology (BIST), Barcelona, Spain; ^2^CBMN (UMR 5248), Centre National de la Recherche Scientifique – IPB, Institut Européen de Chimie et Biologie, University of Bordeaux, Pessac, France; ^3^Laboratoire de Physico-Chimie Moléculaire des Protéines Membranaires (UMR 7099), Université Paris-7 – Centre National de la Recherche Scientifique, Institut de Biologie Physico-Chimique, Paris, France

**Keywords:** Alzheimer's disease, amphipols, amyloid-β, antigen, membrane, oligomers

## Abstract

We have recently reported on the preparation of a membrane-associated β*-barrel Pore-Forming* Aβ42 Oligomer (β*PFO*_*Aβ*42_). It corresponds to a stable and homogeneous Aβ42 oligomer that inserts into lipid bilayers as a well-defined pore and adopts a specific structure with characteristics of a β-barrel arrangement. As a follow-up of this work, we aim to establish β*PFO*_*Aβ*42_'s relevance in Alzheimer's disease (AD). However, β*PFO*_*Aβ*42_ is formed under dodecyl phosphocholine (DPC) micelle conditions—intended to mimic the hydrophobic environment of membranes—which are dynamic. Consequently, dilution of the β*PFO*_*Aβ*42_/DPC complex in a detergent-free buffer leads to dispersion of the DPC molecules from the oligomer surface, leaving the oligomer without the hydrophobic micelle belt that stabilizes it. Since dilution is required for any biological test, transfer of β*PFO*_*Aβ*42_ from DPC micelles into another hydrophobic biomimetic membrane environment, that remains associated with β*PFO*_*Aβ*42_ even under high dilution conditions, is a requisite for the validation of β*PFO*_*Aβ*42_ in AD. Here we describe conditions for exchanging DPC micelles with amphipols (APols), which are amphipathic polymers designed to stabilize membrane proteins in aqueous solutions. APols bind in an irreversible but non-covalent manner to the hydrophobic surface of membrane proteins preserving their structure even under extreme dilution conditions. We tested three types of APols with distinct physical-chemical properties and found that the β*PFO*_*Aβ*42_/DPC complex can only be trapped in non-ionic APols (NAPols). The characterization of the resulting β*PFO*_*Aβ*42_/NAPol complex by biochemical tools and structural biology techniques allowed us to establish that the oligomer structure is maintained even under high dilution. Based on these findings, this work constitutes a first step towards the *in vivo* validation of β*PFO*_*Aβ*42_ in AD.

## Introduction

Amyloid-β (Aβ) oligomers have been proposed as the Aβ species responsible for the neurotoxicity observed in Alzheimer's disease (AD) (Haass and Selkoe, 2007). However, the term Aβ oligomer is vague, as it includes a range of species with distinct stoichiometries and structures that evolve over time. This heterogeneity and transient nature have prevented a consensus as per the specific Aβ oligomer form responsible for AD neurotoxicity (Benilova et al., 2012). To resolve this issue, many laboratories worldwide have developed *in vitro* conditions to obtain as homogeneous and stable Aβ oligomer preparations as possible with which to subsequently establish the links between the specific features of the Aβ oligomer under study and AD neurotoxicity (Lambert et al., 1998; Galeazzi et al., 1999; Bitan et al., 2003; Barghorn et al., 2005; Jan et al., 2010; Sandberg et al., 2010; O'Malley et al., 2014). To establish such links, two strategies have mainly been used. The first one consists of assessing the neurotoxic effects of the Aβ oligomer under study through cell culture treatment (Lambert et al., 1998; Barghorn et al., 2005; Lacor et al., 2007; Ono et al., 2009; Jan et al., 2010; O'Malley et al., 2014) or intracerebral animal injections (Barghorn et al., 2005; Nicole et al., 2016). The second one involves generating antibodies against the Aβ oligomer of interest to establish the oligomer presence in AD human brain tissue (Barghorn et al., 2005; Lambert et al., 2007; Lasagna-Reeves et al., 2011).

Although most Aβ oligomer reports have focused on studying oligomerization in solution, there is an increasingly number of investigations that indicate that the membrane is a target for monomeric and/or oligomeric Aβ forms (Kotler et al., 2014; Roberts et al., 2017; Shrivastava et al., 2017). Specifically, a large number of studies have shown that interaction of Aβ with the membrane results in the formation of Aβ oligomers that function as pores (Arispe et al., 1993; Lin et al., 2001; Kagan, 2012; Bode et al., 2017). Since such pores would compromise neuronal membrane integrity, the authors of these studies proposed amyloid pore formation as a possible means to explain the neurotoxicity observed in AD. In this context, electrophysiological recordings in lipid bilayers demonstrated the presence of multiple single-channel currents of various conductance levels (Arispe et al., 1993; Hirakura et al., 1999; Lin et al., 2001; Quist et al., 2005) and atomic force microscopy (AFM) images revealed that Aβ incorporates into liposomes as oligomeric pores of different sizes (Lin et al., 2001; Quist et al., 2005). However, despite significant evidence in its favor, the amyloid pore hypothesis has yet to be fully confirmed or refuted. This difficulty arises from the heterogeneous nature of the oligomeric pores, which prevents characterization of their individual structure and functional conductivity properties.

We have recently studied Aβ aggregation in the presence of detergent micelles, conditions intended to mimic the hydrophobic environment of membranes. Notably, throughout this study, we found that by fine tuning the ratio of Aβ concentration to detergent micelle concentration ([Aβ]:[M]), we were able to prepare a sample enriched in a specific Aβ oligomer population. Indeed, under optimized dodecyl phosphocholine (DPC) micelle conditions, we showed that Aβ42 assembles into a stable and homogeneous oligomer that inserts into lipid bilayers as a well-defined pore and adopts a structure with characteristics of a β-barrel arrangement. On the basis of these properties, we named this preparation β-barrel Pore-Forming Aβ42 Oligomer (β*PFO*_*Aβ*42_) (Serra-Batiste et al., 2016).

Having access to such a homogeneous and stable Aβ oligomer preparation, we aimed at establishing its relevance in the context of AD. For instance, by assessing the neurotoxic effects of β*PFO*_*Aβ*42_ through cell culture treatment or intracerebral animal injections and by generating antibodies against β*PFO*_*Aβ*42_ to subsequently determine its presence in AD human brain tissue. However, detergent micelles disperse as water-soluble monomers when the total detergent concentration drops at or below the critical micelle concentration (CMC) of the detergent. Therefore, we expected that dilution of the β*PFO*_*Aβ*42_/DPC complex below the CMC of DPC would lead to the dispersion of the DPC micelles into monomers, leaving the oligomer without the hydrophobic micelle belt that stabilizes it and compromising its structural integrity. Dilution is unavoidable in cell culture or animal brain injections for assessing β*PFO*_*Aβ*42_ neurotoxicity or in the blood and other body fluids for generating antibodies. Therefore, exchanging DPC for another hydrophobic biomimetic membrane environment, that would remain associated with β*PFO*_*Aβ*42_ even under high dilution conditions, is a requisite for the validation of β*PFO*_*Aβ*42_ in AD.

Amphipols (APols) are amphipathic polymers designed to stabilize membrane proteins in aqueous solutions (for recent reviews see Popot et al., 2011; Zoonens and Popot, 2014). These polymers bind to the hydrophobic surface of membrane proteins in a non-covalent manner. However, thanks to their multiple contact points, they exhibit an extremely slow dissociation rate. In the absence of a competing surfactant, this feature makes their association with membrane proteins permanent even at extreme dilutions (Popot et al., 2003; Zoonens et al., 2007; Tribet et al., 2009). Because APols are not strong detergents, they can be used to deliver membrane proteins to preformed membranes (Nagy et al., 2001; Pocanschi et al., 2006; Kyrychenko et al., 2012). Moreover, APols have already been successfully used to present antigens to the immune system. Indeed, it has been shown that the native major outer membrane protein (nMOMP) from *C. trachomatis*—a bacterium responsible for a type of sexually transmitted disease—trapped in APols was a much more efficient vaccine than when solubilized in detergent micelles (Tifrea et al., 2011).

In this paper, we investigated the best conditions for β*PFO*_*Aβ*42_ trapping in APols. We tested three types of APols with different chemical structures: a poly(sodium acrylate) based APol comprising 35% of free carboxylates, 25% of octyl chains and 40% of isopropyl groups (A8-35) (Tribet et al., 1996); a derivative from A8-35 in which isopropyl groups were replaced by taurine moieties generating sulfonated APol (SAPol) (Dahmane et al., 2011); and a non-ionic glucosylated APols (NAPols) (Sharma et al., 2012). We found that the integrity of β*PFO*_*Aβ*42_ can only be preserved in NAPols. Characterization of the resulting sample, β*PFO*_*Aβ*42_/NAPol complex, by biochemical tools and structural biology techniques allowed us to establish that the oligomer stoichiometry and structure are maintained after trapping as well as after extensive dilution. Based on the properties of APols, we expect that the β*PFO*_*Aβ*42_/NAPol complex will be an appropriate delivery system to determine β*PFO*_*Aβ*42_ neurotoxic effects and a high quality antigen for the generation of antibodies specific to the β*PFO*_*Aβ*42_ structure. To summarize, transferring β*PFO*_*Aβ*42_ from DPC in NAPols without altering its oligomeric structure is a first necessary step towards the *in vivo* validation of β*PFO*_*Aβ*42_ in AD.

## Materials and methods

### Reagents

DPC was purchased from Avanti Polar Lipids. Dodecyl maltoside (DDM) and A8-35 were acquired from Anatrace. Sulfonated APols (SAPols) and non-ionic APols (NAPols) were synthesized as reported in Dahmane et al. (2011) and Sharma et al. (2012), respectively. Deuterated reagents were obtained from Cambridge Isotope Laboratories. All other reagents were supplied by Sigma-Aldrich unless otherwise stated.

### Preparation of monomeric Aβ42

Aβ42 and Met^35^-[^13^CH_3_]-labeled Aβ42 were synthesized and purified by Dr. James I. Elliott (New Haven, CT, USA). Aβ42 and Met^35^-[^13^CH_3_] Aβ42 in a monomeric state were obtained using size exclusion chromatography (SEC) as described in Serra-Batiste et al. (2016). Briefly, Aβ peptide was dissolved in 6.8 M guanidine thiocyanate (Gdn·SCN) (Life Technologies) at 8.5 mg/mL, sonicated for 5 min in a water bath heated at around 45°C, and diluted to 5 mg/mL of peptide and 4 M Gdn·SCN with H_2_O. It was then centrifuged at 10,000 × g for 6 min at 4°C. The supernatant was injected into a HiLoad Superdex 75 prep grade column (GE Healthcare). The column had been previously equilibrated with 50 mM ammonium carbonate and was eluted at 4°C at a flow rate of 1 mL/min. The peak attributed to monomeric Aβ was collected, and its peptide concentration was determined by High Performance Liquid Chromatography coupled to Photodiode Array Detector (HPLC-PDA). Aliquots at the required amounts were prepared, freeze-dried, and kept at −20°C until use for reconstitution into detergent micelles.

### Quantification of Aβ peptide

The concentration of monomeric Aβ was determined by HPLC-PDA (Waters Alliance 2695 equipped with 2998 photodiode array detector). HPLC-PDA analysis was done using a Symmetry 300 C4 column (4.6 × 150 mm, 5 μm, 300 Å; Waters) at a flow rate of 1 mL/min and a linear gradient from 0 to 60% B in 15 min (A = 0.045 % trifluoroacetic acid (TFA) in water, and B = 0.036 % TFA in acetonitrile) at 60°C. A calibration curve was generated based on an Aβ42 solution that had previously been quantified by amino acid analysis.

### Preparation of βPFO_Aβ42_

We have previously shown that β*PFO*_*Aβ*42_ forms at pH 7.4—consistent with its potential formation under physiological conditions—and at pH 9.0 (Serra-Batiste et al., 2016). However, since β*PFO*_*Aβ*42_ was found to be more stable under the latter pH, we established pH 9.0 as our standard conditions for β*PFO*_*Aβ*42_ preparation. β*PFO*_*Aβ*42_ was prepared by directly dissolving appropriate amounts of freeze-dried monomeric Aβ42 aliquots with 10 mM Tris·HCl pH 9.0 containing 5.5 mM DPC to reach a final Aβ42 concentration of 150 μM. Afterwards, samples were incubated at 37°C for 24 h.

### Selection of the most suitable type of APol for βPFO_Aβ42_ trapping

Three types of APols were used to transfer β*PFO*_*Aβ*42_ from DPC to APols: A8-35, SAPols and NAPols. In each case, appropriate amounts of each APol, from a stock solution at 100 mg/mL prepared in water, were added to the β*PFO*_*Aβ*42_*/DPC* sample to reach Aβ/APol ratios of 1:0.5, 1:1 and 1:2 (w/w). After addition of APol, the sample was gently shaken at 37°C for 20 min in a vortex allowing the formation of the β*PFO*_*Aβ*42_/DPC/APol ternary complex. Vortex shaking was not intended to affect the morphology of the oligomer. Indeed, these are standard conditions extensively used in the literature to transfer membrane proteins from detergent conditions to APols (Zoonens et al., 2005). After vortex shaking, DPC was removed by adding Bio-Beads (Bio-Rad) at a 1:50 (w/w) DPC/Bio-Beads ratio. The samples were incubated at 4°C for 30 min on a wheel. Finally, Bio-Beads were removed by centrifugation. To determine the stability of the β*PFO*_*Aβ*42_/APol complex after DPC removal, samples were analyzed immediately and also after 24 h incubation at 37°C.

### Trapping of βPFO_Aβ42_ in NAPols

The β*PFO*_*Aβ*42_/DPC complex was trapped in NAPols using 1:2, 1:4, and 1:8 (w/w) Aβ42/NAPol ratios following the same protocol as described in section “*Selection of the most suitable type of APol for* β*PFO*_*Aβ*42_ *trapping*.” After DPC removal, the samples were incubated for 24 h at 37°C in order to determine the stability of the β*PFO*_*Aβ*42_/NAPol complex. Only when indicated in the paper and when more extensive detergent removal was required, after DPC removal with Biobeads, three additional dilution/concentration steps were performed. These consisted of a 10-fold dilution of the β*PFO*_*Aβ*42_/NAPol complex by addition of a 10 mM Tris·HCl pH 9.0 solution with 10% D_2_O, followed by a 10-fold concentration of the resulting solution using a Vivaspin 6 (Sigma) centrifugal concentrator device (MWCO 5000 Da). The concentration steps were carried out at 4°C.

### SEC

Samples to be analyzed by SEC were first passed through 0.45-μm filters (Millipore) to remove any insoluble aggregates. Afterwards, 20-μL of each of the samples were loaded onto a Superdex 200 HR 5/150 column (GE Healthcare), eluted at 4°C at a flow rate of 0.5 mL/min and their absorbance was monitored at 220 and 280 nm. For β*PFO*_*Aβ*42_ controls, we loaded β*PFO*_*Aβ*42_/DPC samples onto a Superdex 200 HR 5/150 column equilibrated with 10 mM Tris·HCl, and 100 mM NaCl at pH 9 with and without 0.36 mM dodecyl maltoside (DDM) (Anatrace). The β*PFO*_*Aβ*42_/APol samples were loaded onto a Superdex 200 HR 5/150 column previously equilibrated with 10 mM Tris·HCl and 100 mM NaCl at pH 9.

### Thioflavin T (ThT) fluorescence measurements

ThT fluorescence measurements were carried out on four 150 μM Aβ42 samples prepared as follows: (1) Aβ42 alone: freeze-dried monomeric Aβ42 aliquots were directly dissolved with 10 mM Tris·HCl pH 9.0. (2) Aβ42/NAPol: freeze-dried monomeric Aβ42 aliquots were directly dissolved with 10 mM Tris·HCl pH 9.0 containing NAPol such as the Aβ42/NAPol mass ratio was 1:8. (3) β*PFO*_*Aβ*42_/DPC: freeze-dried monomeric Aβ42 aliquots were directly dissolved with 10 mM Tris·HCl pH 9.0 containing 5.5 mM DPC as described in section “*Preparation of* β*PFO*_*Aβ*42_”, and (4) β*PFO*_*Aβ*42_/NAPol: after β*PFO*_*Aβ*42_/DPC formation, the complex was trapped in NAPols using an Aβ42/NAPol mass ratio of 1:8 following the same protocol as described in section “*Selection of the most suitable type of APol for* β*PFO*_*Aβ*42_ *trapping*.” All samples were incubated for 62 h at 37°C. The definition of the starting point (t_0_) was at the time of Aβ42 resuspension for Aβ42, Aβ42/NAPol, and β*PFO*_*Aβ*42_/DPC samples and following aspiration of the Bio-Beads for the β*PFO*_*Aβ*42_/NAPol sample. After resuspension, samples were kept on ice at all times when possible. After sample preparation, the pH of all samples was adjusted to pH 9.0 and all samples were supplemented with 50 μM ThT, which had been previously dissolved at 2 mM in 10 mM Tris pH 9, filtered (0.2 μm) and chilled to 4°C. While keeping the samples on ice, 100 μL aliquots were added (in triplicate) to a half-area 96 well plate (Corning), which was then immediately inserted into a TECAN Infinite M1000 Pro fluorimeter. Measurements were made every 6 min, although only plotted for every hour, over 62 h at 37°C, using an excitation filter of 450 nm and an emission filter of 486 nm, both with 5 nm bandwidths. Since β*PFO*_*Aβ*42_*/DPC* formation is carried out under quiescent conditions. To best mimic these conditions, samples were not shaken during the ThT assay.

### Negative-staining transmission electron microscopy (TEM)

Negative-staining TEM was carried out for four samples Aβ42, Aβ42/NAPol, β*PFO*_*Aβ*42_/DPC, and β*PFO*_*Aβ*42_/NAPol. All samples were prepared as described in the section *Thioflavin T (ThT) Fluorescence Measurements* and left incubating for 24 h at 37°C. Samples were diluted to 15 μM prior to their visualization using 10 mM Tris·HCl pH 9.0 except for β*PFO*_*Aβ*42_/DPC that was diluted with the same buffer supplemented with 1.5 mM DPC. 5 μL of each sample was deposited for 1 min on carbon-coated copper grids, which had been glow discharged (ELMO, Cordouan Technologies). After a brief wash in uranyl formate, samples were stained with 0.75% uranyl formate for 1 min and dried with filter paper. Grids were observed with a FEI Tecnai F20 electron microscope and images were acquired with a 4kx4k eagle camera (FEI). Images were analyzed with the ImageJ software package (Version 1.51S) (Schneider et al., 2012).

### Limited proteolysis and SDS-PAGE

One hundred and fifty micromolar Aβ42 samples corresponding to β*PFO*_*Aβ*42_/DPC and to β*PFO*_*Aβ*42_*/*NAPol (prepared at an Aβ42/NAPol ratio of 1:8) complexes were digested with Proteinase K at a protease:Aβ42 molar ratio of 1:50. After incubation of the samples with the protease for 45 min at 37°C, the protease was inhibited by adding 4-(2-aminoethyl)benzenesulfonyl fluoride hydrochloride (AEBSF) (Melford) (1 mM final concentration). Afterwards, 7 μL of the resulting samples (before and after digestion) were mixed with 14 μL of 3X sample buffer (SB) and electrophoresed in 1.5 mm-thick SDS-PAGE gels containing 15 % acrylamide. Gels were run at 80-100 V and stained by Coomassie Blue.

### NMR spectroscopy

^1^H-^13^C HMQC spectra were recorded for four samples. The first sample, representative of a random coil Aβ42 monomeric state, was prepared by dissolving an aliquot of freeze-dried monomeric Met^35^-[^13^CH_3_] Aβ42 at a 150 μM concentration in 9 mM Tris·DCl-d_12_, 1 mM Tris·DCl buffer prepared in 100% D_2_O at pH^*^ 8.6. The second sample, corresponding to Aβ42 in an α-helix monomeric state, was obtained by dissolving an aliquot of freeze-dried monomeric Met^35^-[^13^CH_3_] Aβ42 at a 150 μM concentration in 9 mM Tris·DCl-d_12_, 1 mM Tris·DCl buffer prepared in 100% D_2_O and containing 46.4 mM SDS-d_25_ at pH^*^ 8.6. The third sample, corresponding to β*PFO*_*Aβ*42_ formed in DPC micelles, was prepared by first dissolving an aliquot of freeze-dried monomeric Met^35^-[^13^CH_3_] Aβ42 at a 150 μM concentration in 9 mM Tris·DCl-d_12_, 1 mM Tris·DCl buffer prepared in 100% D_2_O and containing 5.5 mM DPC-d_38_ at pH^*^ 8.6. This sample was analyzed after immediate preparation and after 37°C for 24 h. A fourth sample, corresponding to the β*PFO*_*Aβ*42_/NAPol complex, was prepared using the above mentioned β*PFO*_*Aβ*42_ sample followed by trapping in NAPols using an Aβ42/NAPol ratio of 1:8. The spectral window used to acquire these spectra was 5 ppm (^1^H dimension) and 9 ppm (^13^C dimension). ^1^H-^13^C HMQC spectra were measured at 37°C on a Bruker 600 MHz spectrometer equipped with a cryogenic probe head, and data were processed and analyzed using TopSpin software from Bruker. ^31^P spectra were recorded for β*PFO*_*Aβ*42_*/*DPC complex and for β*PFO*_*Aβ*42_*/*NAPol (prepared at an Aβ42/NAPol ratio 1:8) complex at different stages of DPC removal (Figure S1). The spectral window used to acquire ^31^P was 159.53 ppm. A trimethyl phosphine/acetone-d_6_ inset in D_2_O was used as external reference for ^31^P experiments. ^31^P spectra were measured at 37°C on a Bruker 600 MHz. All data were processed and analyzed using TopSpin software from Bruker.

### Western blot

One hundred and fifty micromolar Aβ42 samples corresponding to β*PFO*_*Aβ*42_/DPC complex were diluted 32 times in 10 mM Tris·HCl buffer at either pH 7.4 or pH 9 with or without 1.5 mM DPC. The presence of 1.5 mM DPC, corresponding to the CMC of DPC, in the dilution buffer would keep constant the Aβ42/micelle ratio in the sample and would then be expected to preserve the integrity of the β*PFO*_*Aβ*42_*/DPC* complex. 150 μM Aβ42 samples corresponding to the β*PFO*_*Aβ*42_*/*NAPol complex (prepared at an Aβ42/NAPol ratio of 1:8) was diluted 32 times in 10 mM Tris·HCl buffer at either pH 7.4 or pH 9. 10 μL of 3X SB was added to 20 μL of each sample and 25 μL of the resulting solution were loaded for Western Blot analysis. Samples were electrophoresed using the Mini-protean tetracell system (Bio-Rad, Hercules, CA, USA) on 1.5-mm wide 15 % glycerol-polyacrylamide gels, at 80 V. Afterwards, proteins were transferred to a 0.22-μm nitrocellulose membrane (Amersham Protran) at 100 V for 2 h at 4°C. To improve Aβ detection, membranes were rinsed with 200 mL of phosphate-buffered saline (PBS). Afterwards, membranes were microwaved at 650 W for 1.5 min, kept for 3 min in hot PBS, turned, and microwaved again. Next, membranes were washed in TBS containing 0.1% Tween 20 and then blocked overnight at 4°C with 5 % (w/v) blocking milk. Membranes were immunoblotted using 6E10 (Covance) (1:6,000), dissolved in 5% (w/v) blocking milk, left overnight at 4°C, and detected using a secondary anti-mouse horseradish sheep peroxidase-conjugated antibody (GE Healthcare, UK) using the Immobilon ECL chemiluminescence detection system (Millipore Corp, Billerica, MA, USA). The signals were used to impress X-ray films (Super RX Medical X-Ray, Fujifilm), which were developed using a Hyper processor automated film developer (Amersham Pharmacia Biotech).

## Results

### βPFO_Aβ42_ is stable only when trapped into NAPols

**Figure 1:**
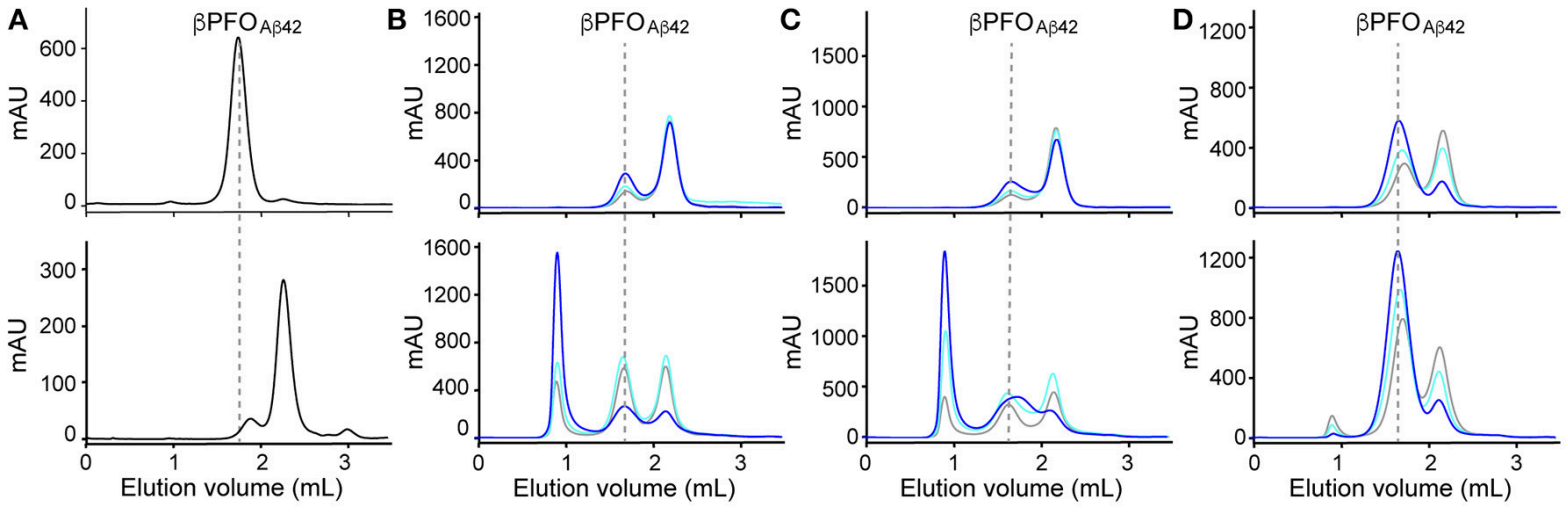
β*PFO*_*Aβ*42_ are stable only when trapped in NAPols. **(A)** SEC of β*PFO*_*Aβ*42_/DPC complex eluted in a column equilibrated with (top) and without (bottom) the presence of detergent in the elution buffer. SEC of β*PFO*_*Aβ*42_trapped in different types of APols: **(B)** A8-35, **(C)** SAPol, and **(D)** NAPol at 1:0.5 (gray line), 1:1 (cyan line) and 1:2 (blue line) Aβ42/APol ratios. After removal of DPC, samples were analyzed immediately (top) and after 24 h of incubation at 37°C (bottom) in a column equilibrated with a detergent- and APol-free buffer. β*PFO*_*Aβ*42_/DPC contains 150 μM nominal Aβ42 concentration in 10 mM Tris, 5.5 mM DPC at pH 9.0 and β*PFO*_*Aβ*42_/APol contains 150 μM nominal Aβ42 concentration trapped at the indicated Aβ42/APol mass ratio in 10 mM Tris at pH 9.0. Experiments have been repeated at least two times and the data shown is representative of them.

As mentioned in the introduction, due to the dynamic nature of detergent micelles, the β*PFO*_*Aβ*42_/DPC complex was not expected to be stable under high dilution conditions. To confirm this expectation, β*PFO*_*Aβ*42_/DPC was analyzed by SEC and the complex eluted either using a detergent-containing buffer (Figure 1A, top) or a detergent-free buffer (Figure 1A, bottom). Under the first conditions, β*PFO*_*Aβ*42_ eluted at 1.8 mL as a major symmetric peak, consistent with the sample comprising an homogeneous population of Aβ42 oligomers (Serra-Batiste et al., 2016) (Figure 1A, top). Instead, under the second set of conditions, which did not contain detergent in the running buffer, we observed a peak with a retention volume at 2.1 mL. Since monomeric Aβ42 elutes at 2.1 mL, this SEC profile is consistent with β*PFO*_*Aβ*42_ oligomer dissociation into monomers and suggests that β*PFO*_*Aβ*42_ requires a hydrophobic environment, such as that provided by a membrane, to be stable (Figure 1A, bottom).

**Figure 2:**
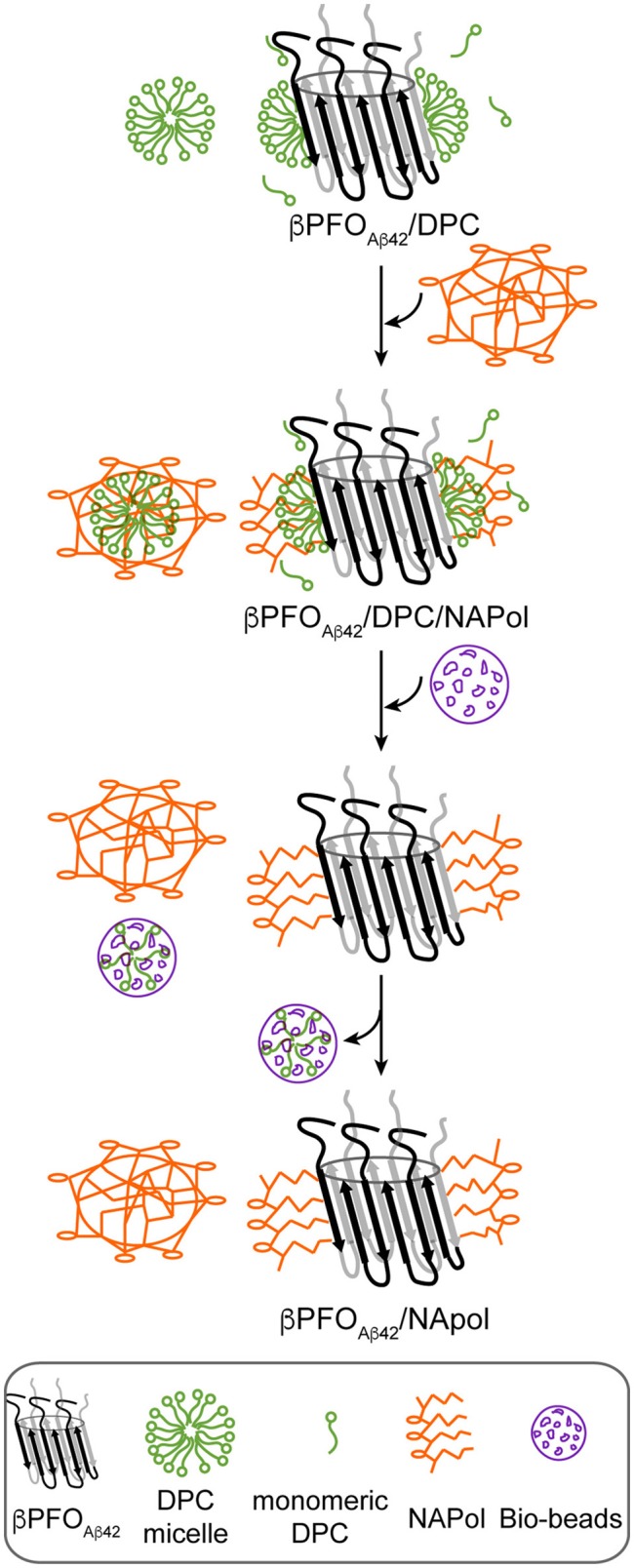
Schematics of the procedure used to trap β*PFO*_*Aβ*42_/DPC in NAPols. NAPol were added to the β*PFO*_*Aβ*42_/DPC complex and the sample was shaken to promote formation of the β*PFO*_*Aβ*42_/DPC/NAPol ternary complex. Afterwards, DPC was removed by adding Bio-Beads. Finally, the DPC/Bio-Beads complex was removed by centrifugation.

Next, we attempted to transfer the β*PFO*_*Aβ*42_ from DPC micelles into another hydrophobic environment that would remain associated with β*PFO*_*Aβ*42_ even under high dilution conditions. To this end, we tested the feasibility of trapping β*PFO*_*Aβ*42_ in three different types of APols: A8-35 (Tribet et al., 1996), SAPols (Dahmane et al., 2011), and NAPols (Sharma et al., 2012). The preparation of β*PFO*_*Aβ*42_/APol complexes is schematically described in the Figure 2. Briefly, the β*PFO*_*Aβ*42_/DPC complex was incubated with the three different types of APols at three Aβ42/APol mass ratios (1:0.5, 1:1, and 1:2). After an incubation period allowing the β*PFO*_*Aβ*42_/DPC/APol ternary complex to form, the concentration of DPC was lowered by adsorption onto polystyrene beads. After DPC removal, the size and homogeneity of the resulting β*PFO*_*Aβ*42_/APol complex was analyzed by SEC, immediately and after 24 h of incubation at 37°C. SEC was carried out on a Superdex 5/150 column equilibrated with detergent- and APol-free buffer. After immediate removal of DPC, all the β*PFO*_*Aβ*42_/APol samples eluted as two peaks at 1.8 mL and 2.1 mL, respectively (Figures 1B–D, top). For samples whose DPC was replaced by A8-35 or SAPols (Figures 1B,C, top), the major SEC peak corresponded to the one eluting at 2.1 mL, assigned to monomeric Aβ42, thus suggesting that these types of APols were not able to efficiently trap the oligomer. Instead, SEC profiles of β*PFO*_*Aβ*42_ trapped in NAPols showed an overall increase in the peak eluting at 1.8 mL, assigned to β*PFO*_*Aβ*42_, as the Aβ42/NAPol mass ratio was increased (Figure 1D, top). This result indicated that NAPols were the best type of APol in which to trap β*PFO*_*Aβ*42_. Next, to determine the stability of the β*PFO*_*Aβ*42_/APol complexes, we analyzed the same samples after 24 h of incubation at 37°C. For samples prepared with A8-35 or SAPols (Figures 1B,C, bottom), a third peak eluting in the void volume was detected. This observation indicates that the samples were not stable and had evolved to higher molecular-weight aggregates. Instead, the SEC profiles of β*PFO*_*Aβ*42_ complexed to NAPols remained stable, since only a small peak eluting in the void volume was detected (Figure 1D, bottom). Notably, β*PFO*_*Aβ*42_/NAPol samples incubated at 37°C for 24 h showed a reproducible increase in intensity for the 1.8 mL peak, assigned to β*PFO*_*Aβ*42_, when compared to the same samples analyzed after immediate removal of the DPC (compare Figure 1D bottom and top). We attribute this increase in intensity to a structural reorganization of β*PFO*_*Aβ*42_/NAPol during incubation. All together, these results indicate that NAPols are the most suitable type of APol in which to stabilize β*PFO*_*Aβ*42_ (Figure 2). They protect β*PFO*_*Aβ*42_ from monomer dissociation and subsequent aggregation. Indeed, NAPols can trap β*PFO*_*Aβ*42_ into a stable complex that shows minimal evolution into higher order aggregates.

**Figure 3:**
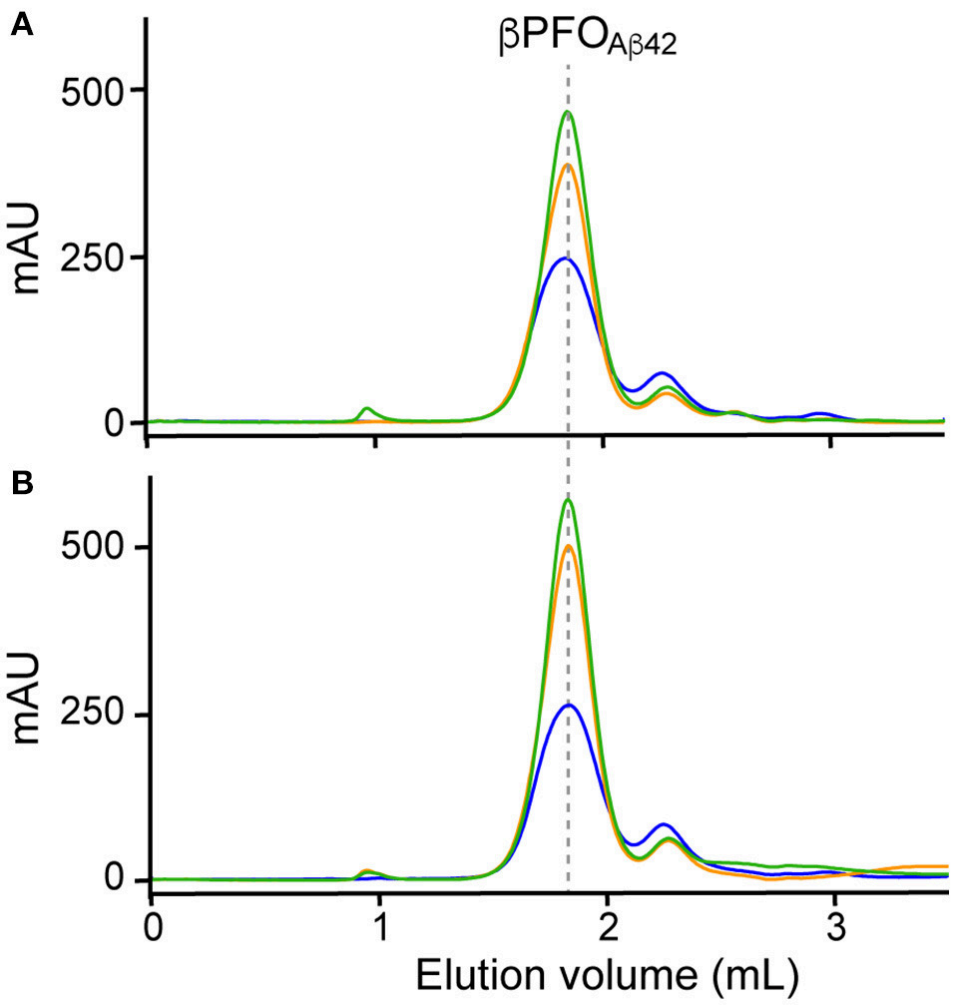
Optimization of trapping conditions of β*PFO*_*Aβ*42_ in NAPols. SEC of β*PFO*_*Aβ*42_ trapped in NAPols at 1:2 (blue line), 1:4 (orange line) and 1:8 (green line) Aβ42/NAPol ratios in a column equilibrated with a detergent-free buffer. After DPC removal, samples were analyzed **(A)** immediately and **(B)** after 24 h incubation at 37°C. β*PFO*_*Aβ*42_/NAPol contains 150 μM nominal Aβ42 concentration trapped at the indicated Aβ42/NAPol mass ratio in 10 mM Tris at pH 9.0. Experiments have been repeated at least three times and the data shown is representative of them.

Finally, since working at the highest Aβ42/NAPol mass ratio of 1:2, we still detected a peak at 2.1 mL, assigned to Aβ42 monomers, we decided to explore higher Aβ42/NAPol mass ratios to increase the overall yield of β*PFO*_*Aβ*42_/NAPol complex formation (Figure 3). To this end, we performed trapping experiments using Aβ42/NAPol mass ratios of 1:2, 1:4, and 1:8. After DPC removal, samples were analyzed by SEC immediately (Figure 3A) and after incubation for 24 h at 37°C (Figure 3B). This analysis revealed that the peak eluting as monomer had slightly decreased in intensity when working at the highest Aβ42/NAPol mass ratios of 1:4 and 1:8. Moreover, since the overall intensity of the peak corresponding to the β*PFO*_*Aβ*42_/NAPol complex was higher when using an Aβ42/NAPol mass ratio of 1:8, we established these conditions as optimal for the trapping of β*PFO*_*Aβ*42_ in NAPols.

### Formation of β*PFO*_Aβ42_/DPC or β*PFO*_Aβ42_/NAPol halts Aβ42 fibrillization

**Figure 4:**
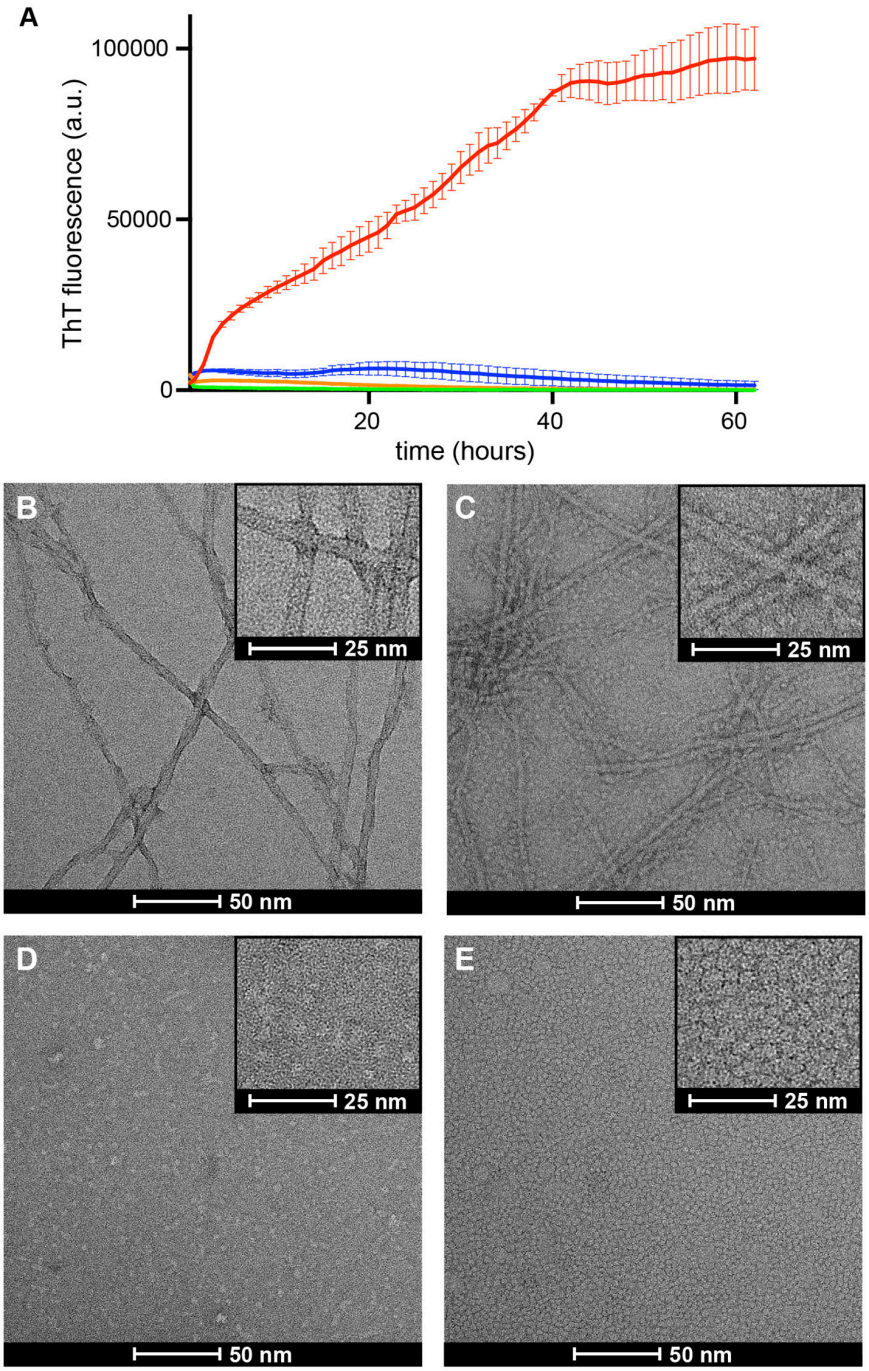
Formation of β*PFO*_*Aβ*42_/DPC or β*PFO*_*Aβ*42_/NAPol halts Aβ42 fibrillization. **(A)** ThT binding assay for Aβ42 alone (red line), Aβ42/NAPol (blue line), β*PFO*_*Aβ*42_/DPC (green line) and β*PFO*_*Aβ*42_/NAPol (orange line) samples incubated at 37°C for 62 h. Electron micrographs obtained for **(B)** Aβ42, **(C)** Aβ42/NAPol, **(D)** β*PFO*_*Aβ*42_/DPC, and **(E)** β*PFO*_*Aβ*42_/NAPol samples after 24 h incubation at 37°C. Samples were prepared as described in the sections Thioflavin T (ThT) Fluorescence Measurements and Negative-Staining Transmission Electron Microscopy (TEM).

In solution Aβ42 has a strong tendency to aggregate into amyloid fibrils. The ThT fluorescence assay is the most well established assay to monitor this process. This assay relies on the capacity of the ThT dye to bind amyloid fibrils. Upon fibril binding, the fluorescence properties of the dye change, thus allowing monitoring of fibril formation. A process characterized by an initial lag phase, followed by a growth phase that leads to a plateau phase when complete fibril formation is reached. Previous studies have shown that when a set of conditions promote formation of a stable membrane-associated amyloid intermediate, the observed lag phase during fibril formation is either increased or completely halted (Rodriguez Camargo et al., 2017). To assess the degree of fibrilization in the samples under study, we monitored ThT fibril formation for Aβ42, Aβ42/NAPol, β*PFO*_*Aβ*42_/DPC, and β*PFO*_*Aβ*42_/NAPol at 37°C (Figure 4A). Incubation of Aβ42 alone, in the absence of DPC micelles or NAPol, exhibited a ThT profile indicative of the formation of abundant amyloid fibrils. Incubation of Aβ42/NAPol, incubation of Aβ42 in the presence of the same NAPol concentration as that used to trap β*PFO*_*Aβ*42_/NAPol, showed a slight increase in ThT fluorescence. In contrast, Aβ42 incubation in the presence of DPC, conditions leading to the formation of β*PFO*_*Aβ*42_/DPC, and incubation of the trapped β*PFO*_*Aβ*42_/NAPol sample did not show any significant increase in ThT fluorescence.

To confirm the results obtained with the ThT fluorescence assay and to learn about the morphology of the samples under study, we analyzed the same samples monitored by ThT fluorescence, after 24 h incubation at 37°C, by TEM. In accordance with the ThT results, the Aβ42 sample incubated alone, showed the presence of abundant amyloid fibrils (Figure 4B). In contrast, although the Aβ42/NAPol sample exhibited only a slight increase in ThT fluorescence, TEM images of this sample revealed the formation of abundant amyloid fibrils, to the same extent as that observed for the Aβ42 sample incubated alone. In addition, the NAPol particles present in this sample appeared to interact with the surface of the fibril (inset of Figure 4C). ThT has been proposed to bind to the surface grooves created by aligned side chains in the fibril axis (Reinke and Gestwicki, 2011). Therefore, one possibility to explain the low ThT fluorescence for the Aβ42 sample incubated in the presence of NAPol would be the binding of NAPol particles at the surface of the fibrils preventing ThT from reaching the fibril surface grooves. In agreement with the ThT results, β*PFO*_*Aβ*42_/DPC and β*PFO*_*Aβ*42_/NAPol samples did not show the presence of any amyloid fibril. Indeed, images corresponding to β*PFO*_*Aβ*42_/DPC show the presence of small spherical objects as well as elongated ones of less than 10 nm in length (Figure 4D). Preparation of the samples for TEM analysis requires a step of washing and staining with a solution of uranyl formate, which do es not contain any detergent. During these steps, the β*PFO*_*Aβ*42_/DPC sample is diluted below the CMC of DPC, leading to the dispersion of the DPC micelles into monomers. This process would leave the oligomer without the hydrophobic micelle belt that stabilizes it, and induce most likely some heterogeneity in the sample. Therefore, the morphology of the objects observed in images obtained from the β*PFO*_*Aβ*42_/DPC sample after staining may not represent the morphology of β*PFO*_*Aβ*42_ in solution. In contrast, NAPol should preserve the structure of β*PFO*_*Aβ*42_ even under high dilution conditions. Indeed, TEM images of the β*PFO*_*Aβ*42_/NAPol sample show a homogenous distribution of spherical objects of about 5 nm in diameter (Figure 4E). Although the size of the objects is very similar to that of a control sample containing only NAPols (Figure S2), it is worth noting that no amyloid fibrils or any other type of aggregates was detected in the images obtained for β*PFO*_*Aβ*42_/NAPol. This observation combined with the SEC analysis of β*PFO*_*Aβ*42_/NAPol (Figure 3) suggests that Aβ42 has to be part of the homogenous spherical objects detected. Altogether, these results indicate that formation of β*PFO*_*Aβ*42_/DPC halts amyloid fibril formation, that previous formation of β*PFO*_*Aβ*42_ in DPC is a requisite for the NAPol to be able to stabilize it, and that the morphology of the β*PFO*_*Aβ*42_/NAPol comprises a homogenous distribution of spherical objects of about 5 nm in diameter.

### NAPols preserve the structure of βPFO_Aβ42_

**Figure 5:**
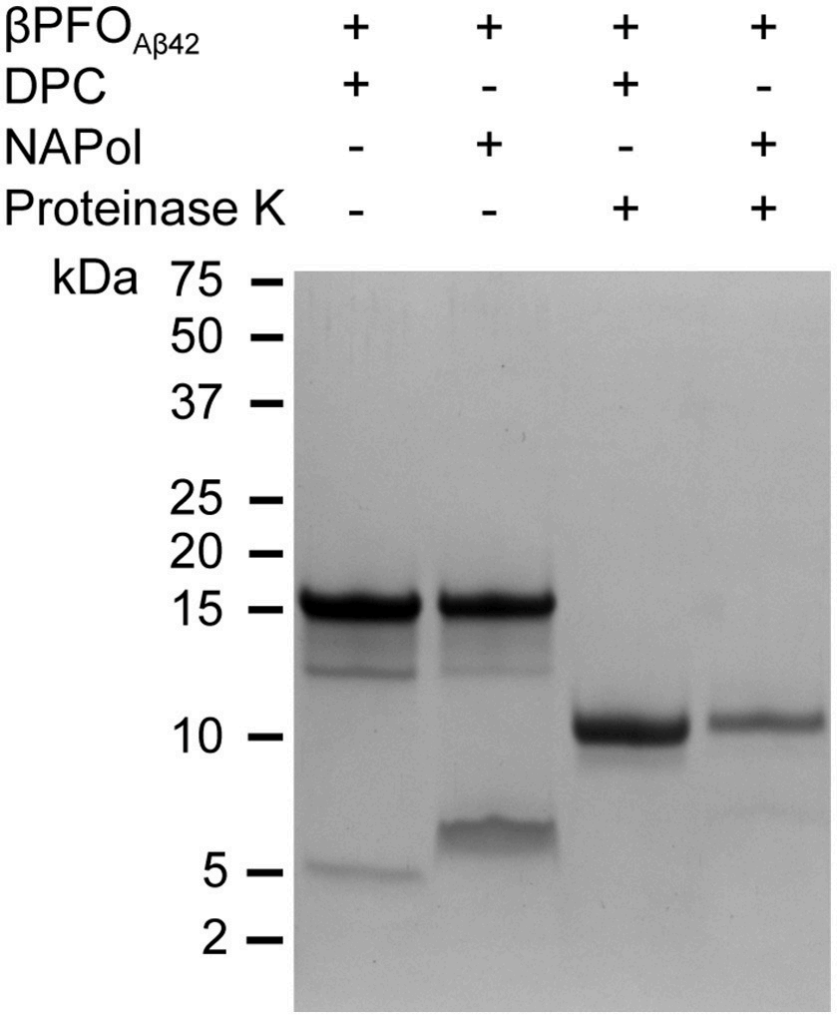
β*PFO*_*Aβ*42_ maintains its structural integrity after trapping in NAPols. SDS-PAGE analysis of β*PFO*_*Aβ*42_/DPC and β*PFO*_*Aβ*42_/NAPol before and after incubation with proteinase K. The β*PFO*_*Aβ*42_/DPC sample contains 150 μM nominal Aβ42 concentration in 10 mM Tris, 5.5 mM DPC at pH 9.0 and the β*PFO*_*Aβ*42_/NApol sample contains 150 μM nominal Aβ42 concentration trapped with a Aβ42/APol mass ratio of 1:8 in 10 mM Tris at pH 9.0. Original version of SDS-PAGE can be found in Figure S3. Experiments have been repeated at least three times and the data shown is representative of them.

Next, we studied whether NAPols preserved specific structural features of β*PFO*_*Aβ*42_. As previously described, β*PFO*_*Aβ*42_ adopts a β-barrel structural arrangement (Serra-Batiste et al., 2016). There are two properties associated with the structure of β-barrel membrane proteins that can be studied by SDS-PAGE analysis. The first is the retention of protein structure upon SDS-PAGE analysis when the sample is not boiled (Otzen and Andersen, 2013). SDS-PAGE analysis of β*PFO*_*Aβ*42_ led to a band at 18 kDa when the sample was not boiled (Figure 5) and to a band at 5 kDa when boiled. These bands are assigned, respectively, to β*PFO*_*Aβ*42_ and monomeric Aβ42 (Serra-Batiste et al., 2016). Moreover, when the β*PFO*_*Aβ*42_ sample was not boiled, apart from the major 18 kDa band, we also detected a lower intensity band at 13 kDa, which suggests the presence of a minor oligomer species within the β*PFO*_*Aβ*42_ preparation. We are currently addressing its nature. It is also worth pointing that although β*PFO*_*Aβ*42_ migrates in SDS-PAGE with the apparent molecular weight of 18 kDa suggestive of a tetramer, this analysis is carried out without boiling the sample to retain β*PFO*_*Aβ*42_ folded structure. Since many β-barrel membrane proteins present different electrophoretic mobilities between the folded and unfolded state (Otzen and Andersen, 2013), β*PFO*_*Aβ*42_ structure may affect its migration on SDS-PAGE, preventing us to interpret the molecular weight of β*PFO*_*Aβ*42_ as a tetramer.

The second property of β-barrel proteins that can be addressed through SDS-PAGE analysis comes from incubation of the protein with proteases. The protease leads to the generation of polypeptide fragments within the solvent-accessible flexible regions of the protein, while leaving the β-barrel intact (Fox and Columbus, 2013). SDS-PAGE analysis of non-boiled β*PFO*_*Aβ*42_ previously incubated with proteinase K led to a lower molecular weight band at 11 kDa (Figure 5), which is consistent with the flexible loops of β*PFO*_*Aβ*42_ being cleaved by the protease (Serra-Batiste et al., 2016). Notably, when the non-boiled β*PFO*_*Aβ*42_/NAPol complexes were analyzed by SDS-PAGE in the absence and in the presence of proteinase K, they mainly ran like the β*PFO*_*Aβ*42_/DPC complex, that is to say as bands of 18 and 11 kDa, respectively (Figure 5). Analysis of the non-boiled β*PFO*_*Aβ*42_/NAPol complex also revealed the presence of a band at around 8 kDa, which we attribute to an anomalous migration of monomeric Aβ in the presence of NAPol. All together, these results indicate that after trapping of β*PFO*_*Aβ*42_ in NAPols, the β-barrel is preserved and the flexible regions within the oligomer remain accessible to the protease.

**Figure 6:**
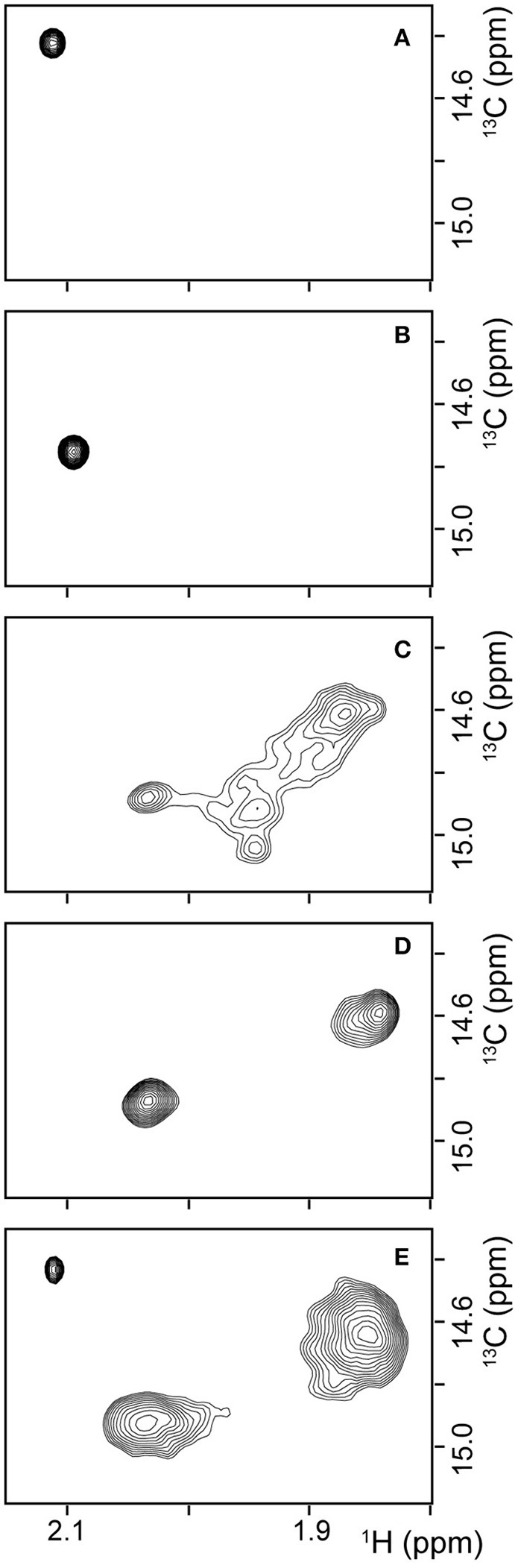
β*PFO*_*Aβ*42_ maintains its structural integrity after trapping in NAPols. ^1^H-^13^C HMQC NMR spectrum of **(A)** a 150 μM Met^35^-[^13^CH_3_] Aβ42 sample dissolved in 9 mM Tris·DCl-d_12_, 1 mM Tris·DCl buffer in 100 % D_2_O at pH* 8.6, **(B)** a 150 μM Met^35^-[^13^CH_3_] Aβ42 sample dissolved in 9 mM Tris·DCl-d_12_, 1 mM Tris·DCl buffer in 100% D_2_O containing 46.4 mM SDS-d_25_ at pH* 8.6, **(C,D)** a Met^35^-[^13^CH_3_] β*PFO*_*Aβ*42_/DPC complex **(C)** after immediate sample preparation and **(D)** after 24 h incubation at 37°C. The β*PFO*_*Aβ*42_/DPC sample contains 150 μM nominal Aβ42 concentration 10 mM Tris, 5.5 mM DPC at pH 9.0, and **(E)** a Met^35^-[^13^CH_3_] β*PFO*_*Aβ*42_/NAPol sample. The β*PFO*_*Aβ*42_/NAPol sample contains 150 μM nominal Aβ42 concentration, trapped with an Aβ42/NAPol mass ratio of 1:8 in 10 mM Tris at pH 9.0. Experiments have been repeated at least three times and the data shown is representative of them.

To obtain additional evidence for the structure of β*PFO*_*Aβ*42_ being preserved when trapped in NAPols, we carried out NMR experiments. In particular, we used Aβ42 samples with the methyl group of the Met 35 side-chain labeled with carbon-13, Met^35^-[^13^CH_3_] Aβ42. These methyl groups are highly dynamic and thus have longer relaxation times than those of most hydrogen and carbon atoms in the protein (Religa et al., 2010). This longer relaxation time allows the application of solution NMR spectroscopy to the study of larger molecular systems through ^1^H-^13^C heteronuclear multiple quantum coherence (HMQC) experiments (Tugarinov et al., 2003). Moreover, since the sequence of Aβ contains a single methionine at residue 35, Met^35^-[^13^CH_3_] Aβ labeling offers the additional advantage of spectral simplification. In addition, we found that the methyl side chain of the Met 35 environment was highly sensitive to changes in the overall structure of the peptide. For example, ^1^H-^13^C HMQC spectra of Met^35^-[^13^CH_3_] Aβ42 dissolved in 10 mM Tris at pH 9—conditions under which the peptide is described to adopt a random coil conformation (Fezoui et al., 2000)—showed a single sharp peak (Figure 6A). Instead, ^1^H-^13^C HMQC spectra of Met^35^-[^13^CH_3_] Aβ42 dissolved in 10 mM Tris, 46.4 mM SDS at pH 9—conditions under which the peptide is described to adopt an alpha-helical structure (Shao et al., 1998)—also showed a single sharp peak but at a different position (Figure 6B). The observation of one peak in ^1^H-^13^C HMQC experiments is indicative of a single average environment for the Met 35 side-chain in the two samples studied. However, the finding that the peaks in each of the samples showed different chemical shifts indicates that the electronic environment surrounding the methionine residue in each sample differs, as would be expected for samples adopting distinct conformations. Next, we used Met^35^-[^13^CH_3_] labeled Aβ42 to prepare β*PFO*_*Aβ*42_ and acquired ^1^H-^13^C HMQC experiments to monitor its formation. After immediate sample preparation, we mainly detected broad peaks (Figure 6C). However, after 24 h incubation at 37°C, they evolved into two sharp and defined peaks with distinct chemical shifts from the previously analyzed samples (Figure 6D). The observation of two defined peaks indicates that the Met 35 side chain perceives two well-defined structural environments that are distinct from those adopted in a random coil and α-helical structure and are characteristic of the β-barrel fold that β*PFO*_*Aβ*42_ adopts (Serra-Batiste et al., 2016).

^1^H-^13^C HMQC spectra of Met^35^-[^13^CH_3_] β*PFO*_*Aβ*42_ trapped in NAPols led to the observation of three peaks: a sharp peak with the same chemical shift as that observed for the spectra of Aβ42 in a random coil conformation (compare Figure 6E to Figure 6A); and two broad peaks with the same chemical shifts as those detected for β*PFO*_*Aβ*42_/DPC (compare Figure 6E to Figure 6D). The observation of the two broad peaks in the ^1^H-^13^C HMQC spectra of the Met^35^-[^13^CH_3_] β*PFO*_*Aβ*42_/NAPols clearly shows that the structure of β*PFO*_*Aβ*42_ is preserved when trapped in NAPols. The broadening of the peaks for the spectra obtained for the β*PFO*_*Aβ*42_/NAPols compared to that of β*PFO*_*Aβ*42_/DPC can be explained by the thicker belt expected for a membrane protein-APol complex compared with that of a membrane protein-detergent complex. Indeed, it has been described that the overall correlation times (τ_c_) of a small membrane protein trapped in APols can be 30–50% longer than that in detergent micelles (Planchard et al., 2014). All together, limited proteolysis and ^1^H-^13^C HMQC NMR experiments indicate that the structure of β*PFO*_*Aβ*42_/NAPol is the same as that of β*PFO*_*Aβ*42_/DPC complex.

### The βPFO_Aβ42_-NAPol complex is stable under high dilution conditions

**Figure 7:**
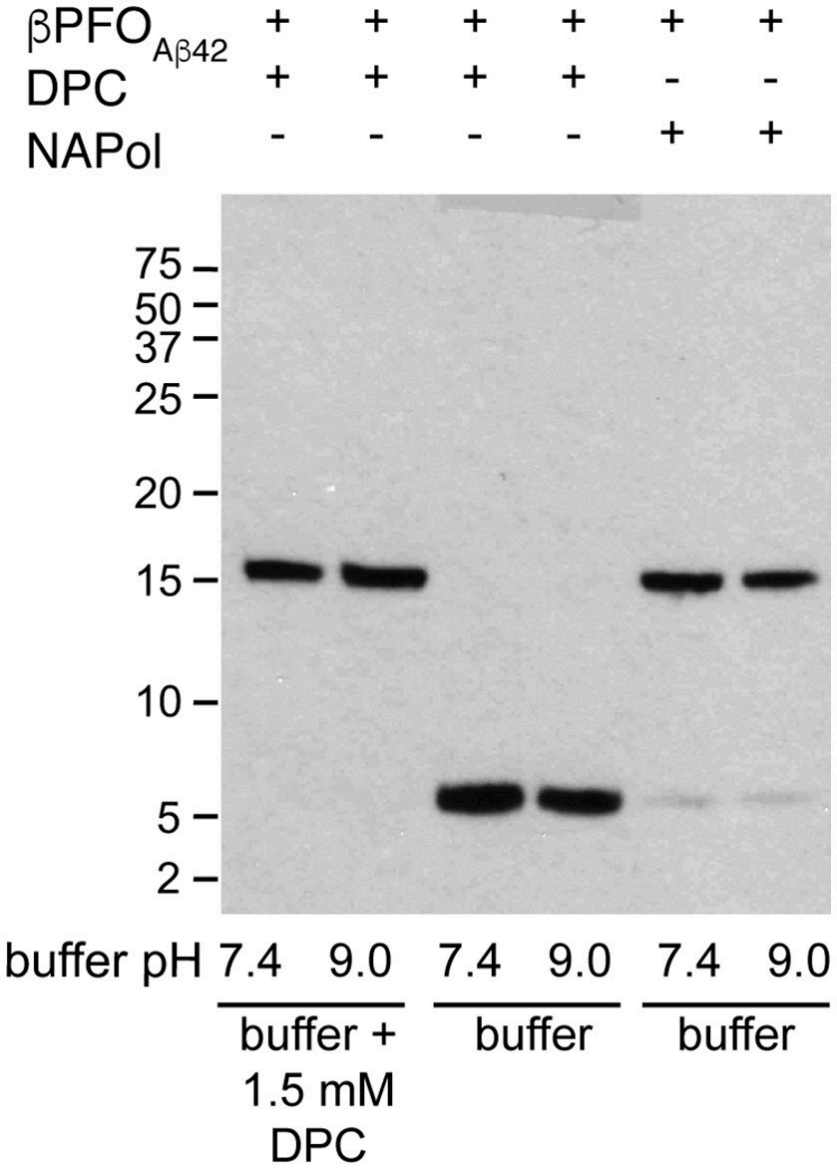
The β*PFO*_*Aβ*42_*/*NAPol complex is stable under high dilution conditions. WB analysis of β*PFO*_*Aβ*42_/DPC complex after dilution at 1/32 in buffer (10 mM Tris at pH 7.4 and pH 9.0) containing or not 1.5 mM DPC and of β*PFO*_*Aβ*42_*/*NAPol complex after dilution at 1/32 in buffer (10 mM Tris at pH 7.4 and pH 9.0). The β*PFO*_*Aβ*42_/DPC sample contains 150 μM nominal Aβ42 concentration in 10 mM Tris, 5.5 mM DPC at pH 9.0 and the sample of β*PFO*_*Aβ*42_/NAPol contains 150 μM nominal Aβ42 concentration trapped with a Aβ42/NAPol mass ratio of 1:8 in 10 mM Tris at pH 9.0. Original version of WB can be found in Figure S3. Experiments have been repeated at least three times and the data shown is representative of them.

An essential property to validate β*PFO*_*Aβ*42_ is that its structure is preserved upon dilution in biological fluids. Therefore, having established that the structure of β*PFO*_*Aβ*42_ is preserved after trapping in NAPols, we aimed to determine whether the structure of the β*PFO*_*Aβ*42_/NAPol complex was also stable under high dilution conditions. To this end, we monitored the integrity of β*PFO*_*Aβ*42_ after extensive dilution (1/32) by WB without boiling the samples. For the β*PFO*_*Aβ*42_/DPC complex to be stable under high dilution conditions, the dilution buffer must contain DPC at its CMC (Figure 7, lanes 1 and 2). Under these conditions, the [Aβ42]:[M_DPC_] ratio in the sample is maintained, allowing the oligomer to remain stable at both physiological pH 7.4 and at pH 9.0. However, when the β*PFO*_*Aβ*42_/DPC complex is diluted in a buffer free of detergent, the oligomer is unstable and breaks down into Aβ42 monomers at both pH values (Figure 7, lanes 3 and 4). In our SDS-PAGE gels, Aβ42 monomers migrate with an apparent molecular weight of 6 kDa, slightly larger than expected. We attribute this result to the fact that to preserve the β*PFO*_*Aβ*42_ β-barrel fold, we do not boil our samples, which may prevent their complete denaturation, and consequently a lack of correlation with the molecular weight of the protein standards. Notably, when the β*PFO*_*Aβ*42_/NAPol complex is diluted in a buffer free of detergent micelles and NAPols, the oligomer is stable at physiological pH 7.4 and at pH 9.0 (Figure 7, lanes 5 and 6). Indeed, only 5.3% of the sample is recovered as monomer. This result indicates that NAPols remain irreversibly attached to β*PFO*_*Aβ*42_, thereby conferring the oligomer protection against extensive dilution and thus making the β*PFO*_*Aβ*42_/NAPol complex an excellent system to establish β*PFO*_*Aβ*42_ functional effects on relevant disease models and to use it as an antigen for the development of conformational specific antibodies against β*PFO*_*Aβ*42_.

## Discussion

**Figure 8:**
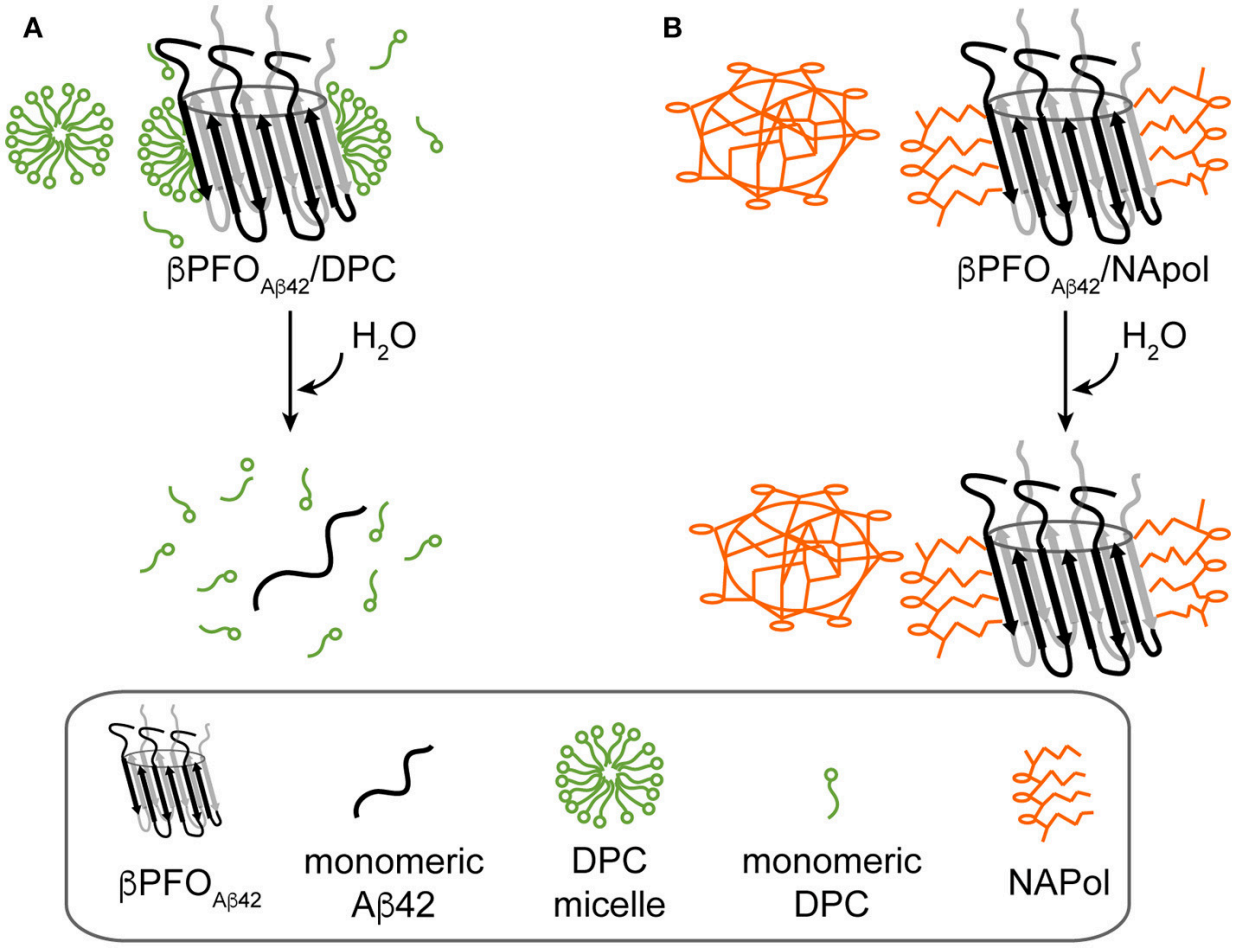
Schematics of the behavior of β*PFO*_*Aβ*42_*/*DPC and β*PFO*_*Aβ*42_*/*NAPol under high dilution conditions in a detergent-free buffer. **(A)** β*PFO*_*Aβ*42_*/*DPC breaks into monomeric Aβ42 and **(B)** β*PFO*_*Aβ*42_*/*NAPol is stable.

We have recently reported on the preparation of β*PFO*_*Aβ*42_, a stable and homogeneous Aβ42 oligomer (Serra-Batiste et al., 2016). Our current aim is to establish its relevance in the context of AD. However, the stability of β*PFO*_*Aβ*42_ relies on the presence of detergent (DPC) micelles in the buffer in which it is diluted (Figure 8A). This requirement limits any biological experiments aiming at establishing the neurotoxicity of this oligomer and/or the generation of conformational specific antibodies. Throughout this work, we have overcome this important limitation. We show that β*PFO*_*Aβ*42_ can be trapped in APols, specifically using NAPol and that the β*PFO*_*Aβ*42_/NAPol complex retains the structure of the oligomer and is stable upon dilution in a detergent- and NAPol-free buffer (Figure 8B). Preservation of the β*PFO*_*Aβ*42_ structure was assessed by comparing the properties of the β*PFO*_*Aβ*42_/DPC complex to those of β*PFO*_*Aβ*42_/NAPol by SDS-PAGE analysis without boiling the sample, in the absence and in the presence of protease K (Figure 5), and by ^1^H-^13^C HMQC experiments (Figure 6). These experiments allowed us to establish that specific structural fingerprints of the β*PFO*_*Aβ*42_/DPC samples were maintained in the β*PFO*_*Aβ*42_/NAPol ones. These fingerprints include the same electrophoretic mobility in an SDS-PAGE without boiling the sample in the absence and the presence of protease K (Figure 5) and detection of peaks at the same ^1^H and ^13^C NMR chemical shift in ^1^H-^13^C HMQC NMR spectra (Figure 6). Finally, preservation of the β*PFO*_*Aβ*42_ structure under high dilution conditions in a detergent- and NAPol-free buffer was assessed by SEC (Figure 1) and by WB analysis without boiling the samples (Figure 7). In this experiment, β*PFO*_*Aβ*42_ was stable only when trapped in NAPols. The stability of β*PFO*_*Aβ*42_/NAPol under high dilution conditions is in agreement with previous reports (Zoonens et al., 2007). For example, after trapping of the transmembrane domain of OmpA (tOmpA) in A8-35, no APol desorption was observed even after extensive dilution (1/1,000) of the complex. This phenomenon was explained by the low critical APol aggregation concentration (CAC), below which the APol particles dissociate (Giusti et al., 2012).

As a first approach to establish the relevance of β*PFO*_*Aβ*42_ in AD, we plan to determine whether β*PFO*_*Aβ*42_ is recognized by other anti-Aβ oligomer antibodies described in the literature (Kayed et al., 2003, 2009; Barghorn et al., 2005; Lambert et al., 2007). The most widely used anti-Aβ oligomer in the literature is A11 (Kayed et al., 2003). A11 has been reported to recognize universal features of various Aβ oligomer preparations, as well as oligomers formed by other amyloid proteins. However, taking into account the properties of β*PFO*_*Aβ*42_, we also plan to prove its immunoreactivity against the anti-annular anti-protofibril (αAPFs) antibody, which apart from recognizing ring-shaped and pore-like structures formed by many different amyloidogenic proteins and peptides, also recognizes heptameric α-hemolysin pores (Kayed et al., 2009) and exhibits intracellular labeling in AD brain-derived tissue (Lasagna-Reeves et al., 2011). Moreover, since all reported anti-Aβ oligomer antibodies have been generated using rather heterogeneous Aβ oligomer preparations, the homogeneity and stability of β*PFO*_*Aβ*42_/NAPols preparation offer us an excellent opportunity to obtain conformation-specific antibodies against a specific Aβ oligomer preparation. By comparing brain immunoreactivity with the already described anti-Aβ oligomers antibodies to that obtained with the anti-β*PFO*_*Aβ*42_ antibodies, we expect to assess whether β*PFO*_*Aβ*42_ is one of the range of Aβ oligomers already described in the literature or constitutes a new class of oligomer. The generation of antibodies against β*PFO*_*Aβ*42_ using β*PFO*_*Aβ*42_/NAPols, could raise the concern of whether NAPols could sterically mask the immunogenic β*PFO*_*Aβ*42_'s epitopes. Limited proteolysis experiments have revealed that the flexible regions within the oligomer trapped in NAPols remain accessible to the protease (Figure 5) suggesting that the β*PFO*_*Aβ*42_/NAPols have accessible epitopes for antibody binding. Moreover, in the context of the work with other membrane proteins, surface plasmon resonance experiments have revealed that immobilized-membrane proteins trapped in APols are recognized by specific antibodies, suggesting a good accessibility of their epitopes (Charvolin et al., 2009; Basit et al., 2012; Giusti et al., 2015).

The physicochemical properties of the APols have been shown to be critical for the successful handling of β*PFO*_*Aβ*42_. Three APols were tested: A8-35, SAPols, and NAPols. The chemical structure of A8-35 comprises 35% free carboxylates, 25% octylamide moieties, and 40% isopropylamide moieties (Popot et al., 2011). Since aqueous solubility of A8-35 depends on the deprotonation of its carboxylate moieties, a process that starts just above pH 7.0, its use is limited to pHs higher than 7.0 (Gohon et al., 2006). SAPols comprise 35% free carboxylates, 25% octylamide moieties, and 40% taurine, the latter comprising sulfonate groups. SAPols have a higher charge density than A8-35 (75 vs. 35%). Moreover, since 40% of the 75% charge density comes from sulfonate groups, which do not protonate at pH 0, SAPols allow working at very low pHs (Dahmane et al., 2011). Finally NAPols correspond to glucosylated, non-ionic APol with a 0% charge density, and they are therefore insensitive to pH (Bazzacco et al., 2012). Because the β*PFO*_*Aβ*42_/DPC complex is more stable at pH 9.0, we tested conditions of trapping in APols at this pH, conditions compatible with the use of all three types of APols. However, only NAPols allowed successful β*PFO*_*Aβ*42_ trapping. The use of charged APols, either A8-35 or SAPols led to sample recovery mainly as monomer, which aggregated as a function of time (Figures 1B,C). The observation of monomer recovered as the main species when using ionic APols could be explained by the incapacity of ionic APols to form a ternary complex with the β*PFO*_*Aβ*42_ oligomer or the incompatibility of the β*PFO*_*Aβ*42_'s structural integrity with a highly charged surfactant in its vicinity. In this situation, upon depletion of DPC, the oligomer would be expected to break down into monomers, as observed when DPC is depleted from the β*PFO*_*Aβ*42_/DPC complex in the absence of APol (Figure 1A, bottom).

The major implication of our work is that β*PFO*_*Aβ*42_/NAPol has the properties to be used as a delivery system to determine β*PFO*_*Aβ*42_ neurotoxic effects and as a high quality antigen suitable for the generation of conformational specific antibodies against β*PFO*_*Aβ*42_. These antibodies will be essential tools to validate the role of β*PFO*_*Aβ*42_ in relevant models of AD. Moreover, having access to the β*PFO*_*Aβ*42_/NAPol complex extends the types of analysis that can be done to further characterize β*PFO*_*Aβ*42_ structure. For example, mass spectrometry (MS) is the main technique through which to establish the stoichiometry of membrane protein complexes. APols have been shown to be compatible with MS analysis as they can be released in the gas phase while conserving supramolecular interactions (Leney et al., 2012; Hopper et al., 2013; Watkinson et al., 2015). Therefore, β*PFO*_*Aβ*42_/NAPol can contribute to establishing β*PFO*_*Aβ*42_ stoichiometry. Moreover, membrane protein/APol complexes are routinely used to determine the 3D structure of membrane proteins by cryo-EM (Bai et al., 2015). Apart from stabilizing the target protein, APols have been shown to spread the particles onto the microscope grids. Therefore, although β*PFO*_*Aβ*42_ is too small to be characterized by this technique, one can envision that a higher molecular weight complex between, for example, β*PFO*_*Aβ*42_/NAPol and an antibody or antibody fragment could be studied by cryo-EM (Wu et al., 2012). In summary, preparation of β*PFO*_*Aβ*42_/NAPol opens a window of opportunities for the further characterization of β*PFO*_*Aβ*42_ including its structural characterization by MS and cryo-EM, for establishing β*PFO*_*Aβ*42_ neurotoxic effects and for the generation of specific antibodies against its structure, which are critical tools to validate the role of β*PFO*_*Aβ*42_ in AD.

## Author contributions

MS-B designed and carried out SEC, SDS-PAGE, NMR, and WB experiments, analyzed the corresponding data, and wrote the manuscript. JT designed and carried out ThT and TEM experiments, and analyzed the corresponding data. FG synthesized SAPol and NAPol. MZ provided conceptual advice on the design of the experiments and revised the manuscript. NC conceived the study, designed the experiments, analyzed the data, and wrote the manuscript.

### Conflict of interest statement

The authors declare that the research was conducted in the absence of any commercial or financial relationships that could be construed as a potential conflict of interest.
