# Supplementary figures and images for: Stabilization of a Membrane-Associated Amyloid-β Oligomer for Its Validation in Alzheimer's Disease
Source: Front Mol Biosci. 2018 Apr 19;5:38. doi: 10.3389/fmolb.2018.00038 (PMC5917194; doi:10.3389/fmolb.2018.00038)

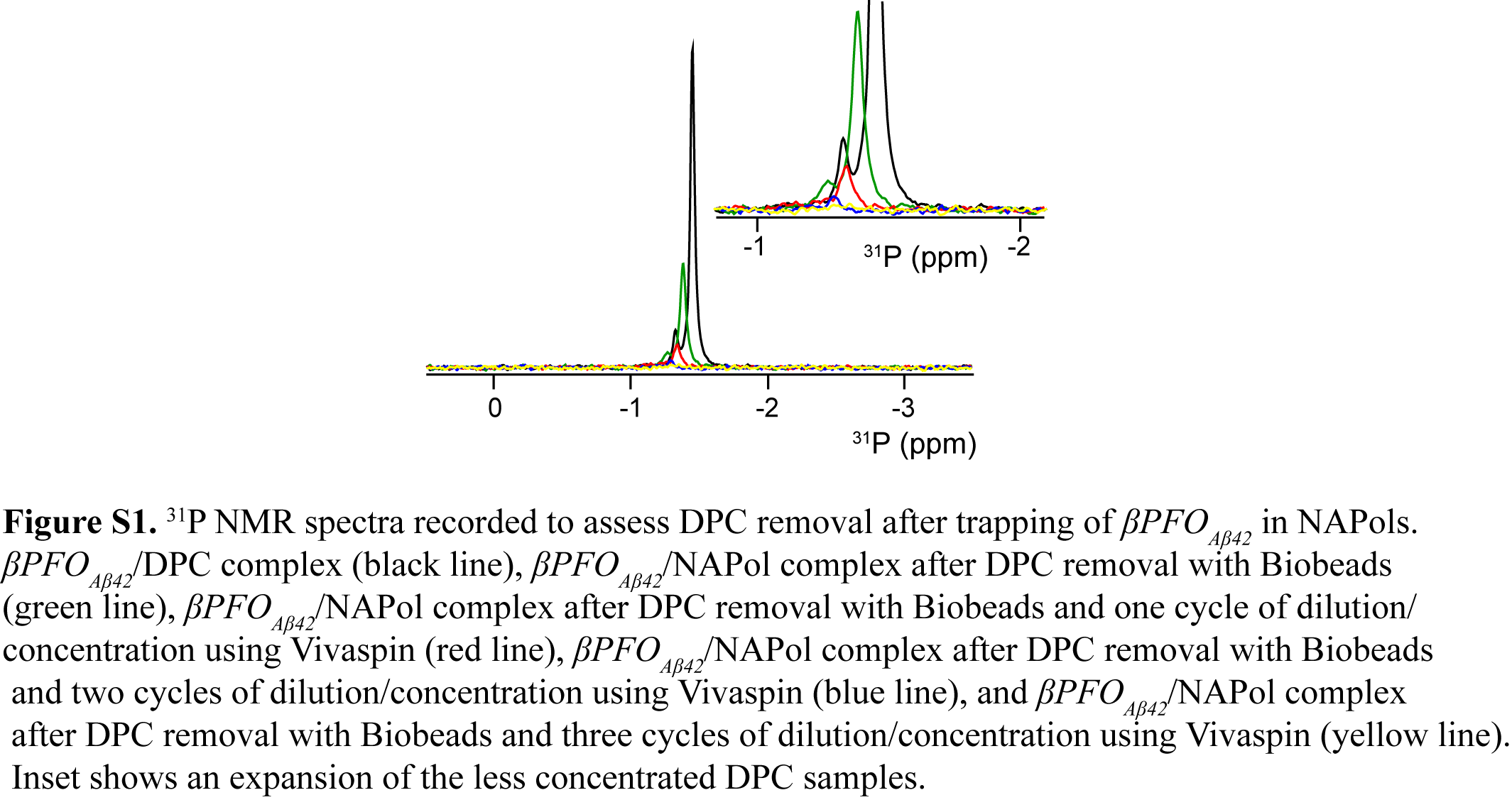

Supplement: Supplementary file 1 [file Image1.TIF]

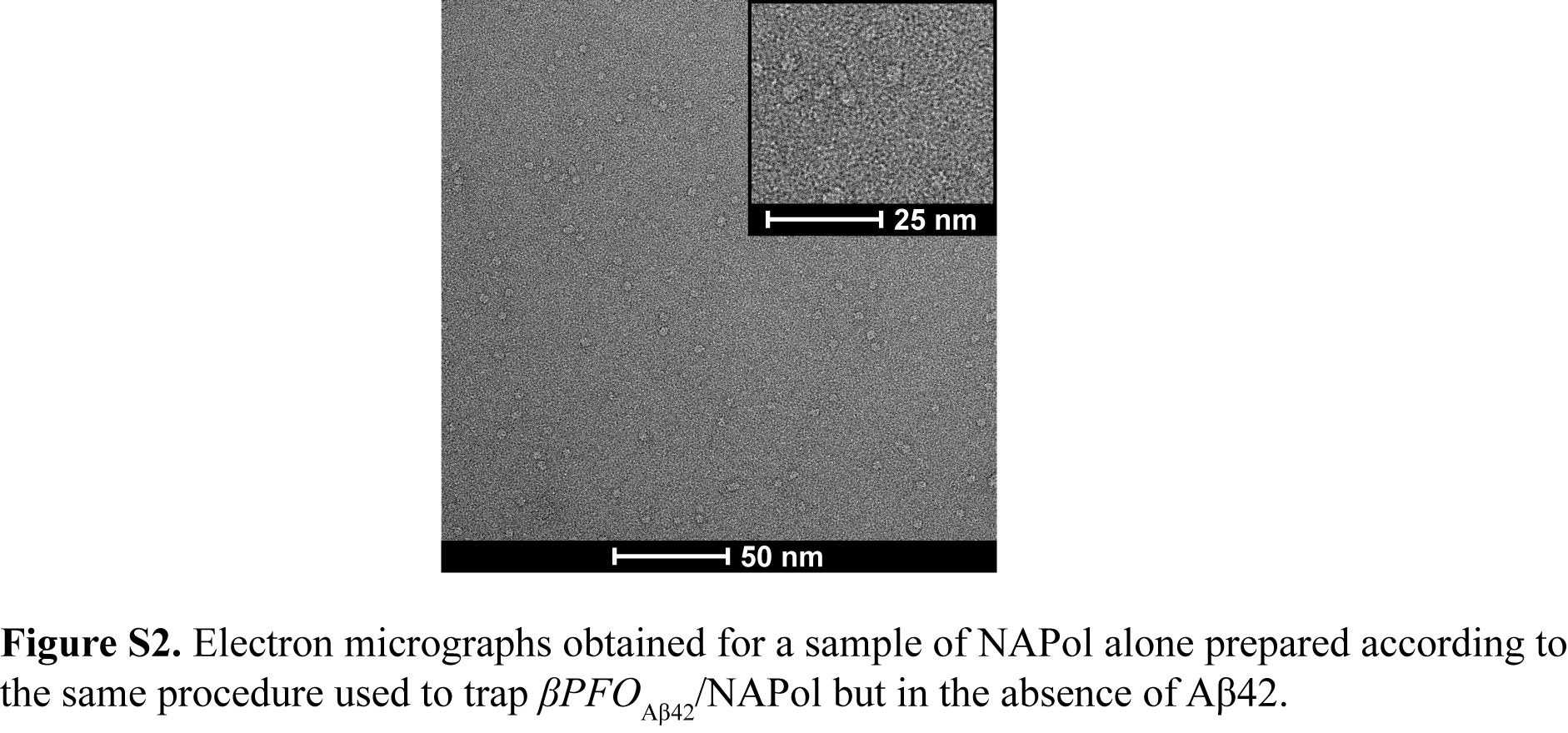

Supplement: Supplementary file 2 [file Image2.TIF]

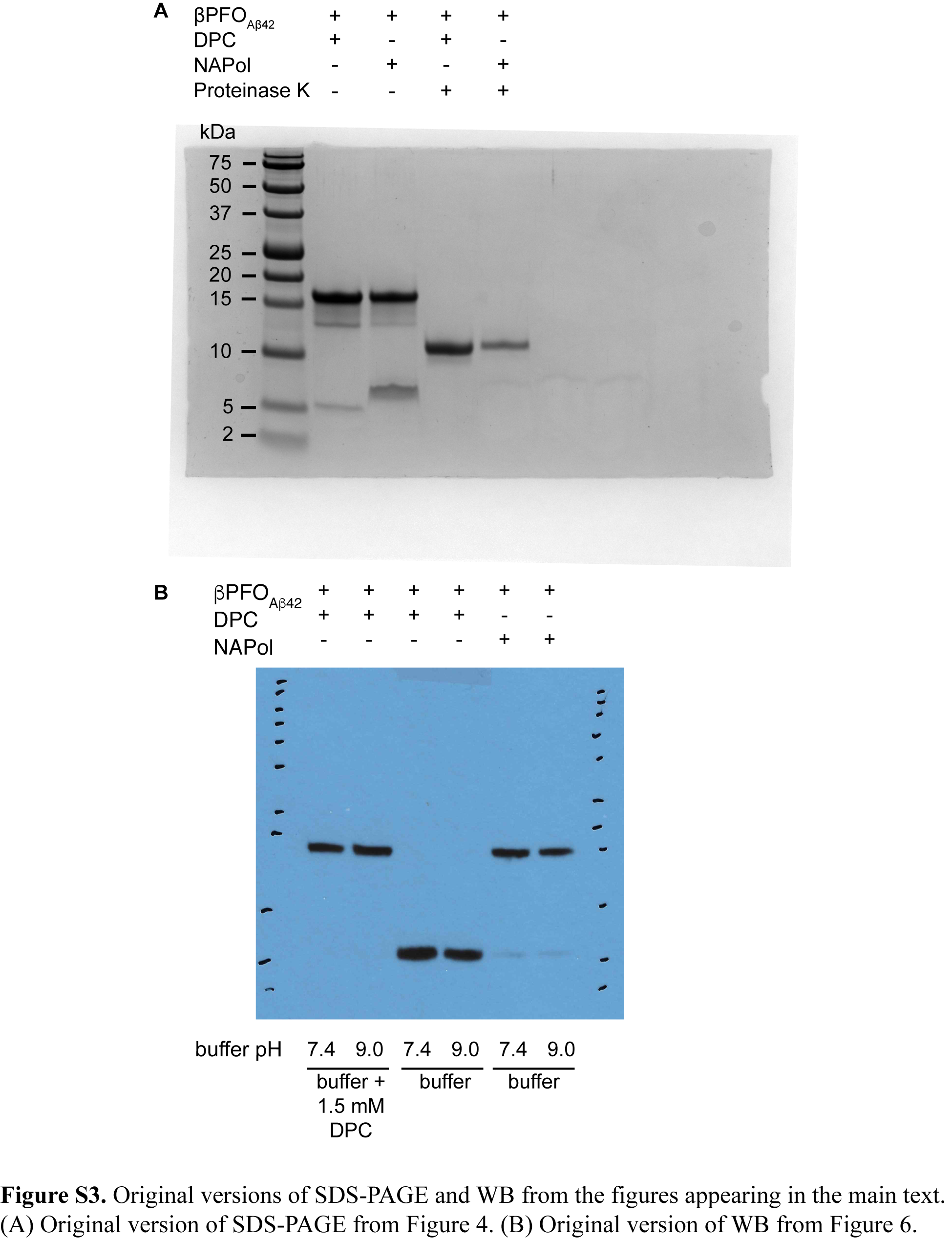

Supplement: Supplementary file 3 [file Image3.TIF]
